# Farmer Health

**Published:** 1863-01

**Authors:** 


### HALL'S JOURNAL OF HEALTH.

Our Legitimate Scope is almost boundless: for whatever begets pleasurable and harmless feelings, promotes Health; and whatever induces disagreeable sensations, engenders Disease.

WE AIM TO SHOW HOW DISEASE MAY BE AVOIDED, AND THAT IT IS BEST, WHEN SICKNESS COMES, TO TAKE NO MEDICINE WITHOUT CONSULTING A PHYSICIAN.

Vol. X.] JANUARY, 1863. [No. 1.

### FARMER HEALTH.

In passing through a lunatic asylum, the visitor is sometimes surprised to learn that the most numerous class of unfortunates are from the farm; and yet in England, in 1860, but one fifth of the population were agricultural. Nor do farmers live the longest. Travelers and natural philosophers average a greater age. The clergyman who devotes his life to study and late hours; who spends three fourths of his existence in-doors; who does not average two hours' muscular exercise in twenty four; who is compelled to an inactivity of body which would seem enough to undermine any constitution, to say nothing of the many depressing influences connected with his office in listening to the troubled, in counseling the sick, and in waiting on the dying and the dead, even he often survives the farmer who rises with the lark to breathe the pure out-door air; whose undisturbed nights; whose supposed independence of the world; upon whose table is daily placed the fresh butter and the new-laid eggs, with pure rich milk from the spring-house, all cool and sweet, vegetables just dug from the ground or pulled from the vine, and melons taken from the garden, berries from the bending bushes, and fruits, luscious and perfect and ripe from the orchard within the hour; in short, a class of men whose entire surroundings of quiet and plenty and independence would seem to guarantee a healthful and happy old age, do not attain it as often as some other classes whose habits and modes of life are not, other things being equal, as favorable to longevity. In the light of these statements, it is proposed to inquire.

First: Why is the farmer more liable to insanity than the citizen? Second: Why does he not average a longer life?

Incessant thinking on any one subject tends to craze the brain; and it does unhinge the intellect of multitudes, as witness the fate of men of "one idea;" of inventors; of inveterate students of prophecy; of those who abandon themselves to thinking of the loved and lost; of the victims of remorse or mortified pride; or of those who feed on sharp-pointed memories. Learned physicians of all civilized countries agree that, in cases like these, it is best to divert the mind, by travel, to a new class of thoughts, to a greater variety of objects of contemplation. It is known that within a short time the attention of the French government has been officially drawn to the fact that one in ten of the young gentlemen who are educated for the army, in the mathematical department, becomes deranged; this is because the mind will not bear exclusive action on one subject. This is the key to the so frequent cases of insanity and suicide among farmers; their subjects of thought are too few; their life is a ruinous routine; there is a sameness and a tameness about it, a paucity of subjects for contemplation, most dangerous to mental integrity.

It is too much the case with our farming population that they have no breadth of view; they can not sustain a conversation beyond a few comments on the weather, the crops, the markets, and the neighborhood news. And it is worthy of note that their remarks on these subjects are uniformly of the complaining and unhopeful kind, as if their occupation and their thoughts were on the same low and depressing level. This is because the mind is not used enough; is not waked up by a lively interest in a sufficient variety of subjects to promote a healthful tone.

The proper and the all-powerful remedy against the sad effects of a plodding, routine existence, is a higher standard of general intelligence and a livelier attention to what is too often derisively styled "book-farming." The highest form of human health is found in those who exercise the brain and the body in something like equal proportions. If the greater share of the nervous energies is sent out through the muscles, they will be largely, even preternaturally, developed; but then the brain languishes for want of its due amount of aliment, vigorous thought, while that same body, having been unduly worked, wears out before its time and prematurely decays. It is even better for the mind and body both, that if either has the larger share of exercise it should be the brain, for thereby the chances of longer life are increased, since statistics clearly show that, as a general rule, the most intellectual live the longest. Prof. Pierce, of Cambridge, after having examined the subject closely in reference to the young gentlemen pursuing their studies at Harvard University, remarks, as the result of his observations, that: "Taking classes in the average, those are the first to die who are the dullest and most stupid; while, as a general rule, those who exercise their brains most constantly, thoroughly, and faithfully, are the longest lived."

The lamented President Felton was accustomed to urge upon the young gentlemen of his classes, with great earnestness, as a means of high health, that they should "use the mind;" use it actively, and on a variety of subjects, so as to avoid any dull routine.

It is an observed fact that many of those sent to penitentiaries for long terms, or for life, become idiotic; but that among the number there is seldom found one who had even small pretensions to a liberal education or to mental culture in any direction. The gifted and unfortunate Mary Queen of Scotts, after lingering eighteen years in prison, came forth to the block with that vigor of mind and clearness of intellect and composure of manner which bespoke a healthful brain. Multitudes of distinguished men have passed a large portion of their lives in prisons, yet maintained their mental integrity, and lived long enough afterwards to accomplish great deeds. Count Confalioneri, having rendered himself obnoxious to the Austrian government, was confined in a dungeon ten feet square for six years, with so dim a light that he could not distinguish the features of the solitary companion of his misfortunes; after which time he remained nine years longer, entirely alone. He writes of himself: "Only one event broke in upon my nine years' vacancy. One day — it must have been a year or two after my companion left me — my dungeon-door was opened, and a voice, I knew not whence, uttered these words: 'By order of his Imperial Majesty, I intimate to you, that one year ago your wife died.' Then the door was shut. I heard no more. They had but flung this great agony in upon me, and left me alone with it again." Without a book, without a companion, without any intelligence from the outer world, confined in a dark dungeon, living on the coarsest food, having those inward resources which a superior education gave, he fed upon them, and thus maintained both mental and bodily health; while the uninstructed farmer, who can feed on the fat of the land, who passes near three fourths of his existence in the blessed sunlight, greedily drinking in the luscious out-door air in all its purity, with no restraint of bodily liberty, so abandons himself to the dull routine which comprises almost nothing but to work and eat and sleep, often finds in a less time than fifteen years, that vigor of mind and health of body are both on the wane. But a better time is coming, through the influence of our glorious Public-School system, when it shall no longer be considered an all-sufficient qualification for a farmer that he have a vigorous frame and intelligence enough to skillfully wield an ax or turn a furrow or drive a team. Men are already beginning to perceive that encouragingly remunerative farming is the reward of those who have made themselves familiar with the analysis of soils, who have some knowledge of botany and vegetable chemistry, who have given some study to ascertain the surest way of obtaining the best seeds and the best breeds, and who have "method in their" book "madness," in the selection of cions and grafts and roots and plants. Such men not only make money by farming, but have a positive delight in their labor, and in waiting for results; for one of the sweetest sensations possible to the human mind is the development of useful practical facts as the result of trials and experiments. If the young farmer then begins life with a better literary education, and every farm-house is regularly visited by some well-conducted agricultural periodical, the mental horizon of the hard-working tiller of the soil will soon become so extended that a demented farmer will become the rarest of sights. There is another item in reference to the farming population of this country, which certainly adds to the number of its lunatics; it is that grim specter, DEBT, which is voluntarily set up in the households of three farmers out of four, whether in the cabin of the thriftless squatter or in the mansion of the princely planter. It is generally a very grave mistake, in the hope of making money by the rise of land, to purchase more than can be conveniently paid for on the spot, or more than can be advantageously cultivated with the force at command. This demon of debt, with its "interest" eating out the farmer's substance ceaselessly and remorselessly, day and night, summer and winter, in sunshine and in shade, is in multitudes of cases a vain sacrifice to the Moloch of gain, a yawning maelstrom, pitiless and inappeasable; it eats out half the joys of many families, by reason of the self-denials, the always losing "make-shifts," the working to disadvantage and consequent extra labor, with those anxieties and solicitudes which are necessarily imposed, and which, in their turn, induce irritation of mind, irascibility of temper, and that forgetfulness of those domestic amenities which many times convert a trouble into a pleasure and alleviate or take entirely away half the burdens of life. These acerbities of temper grow by what they feed upon, and seldom fail in the end to leave an evil impress on the character of those upon whom the disturbing consciousness of debt presses with the weight of the nether millstone, impelling too often to the razor, the river, or the halter; for it is not an unknown thing, by any means, that the hard-working farmer becomes a suicide. The whole subject is presented, with additional thoughts, in the eighth volume of HALL'S JOURNAL OF HEALTH, New-York, page forty-two; to make this article more specifically practical, the attention of farmers' families is invited to the chief and direct causes of nine tenths of the diseases which cloud their happiness, which interfere with their prosperity, and often largely add to discouraging expenditures of the means which it caused so much labor to acquire; and first to

### EATING.

THE stomach has two doors, one for the entrance of the food, on the left side, the other, for its exit, after it has been properly prepared for another process. As soon as the food is swallowed, it begins to go round and round the stomach, so as to facilitate dissolution; just as the melting of a number of small bits of ice is expedited by being stirred in a glass of water; the food, like the ice, dissolving from without, inwards, until all is a liquid mass.

When food is unnaturally detained in the stomach, it produces wind, eructations, fullness, acidity, or a feeling often described as a "weight," or "load," or "heavy." But nature is never cheated. Her regulations are never infringed with impunity; and although an indigestible article may be allowed to pass out of the stomach, it enters the bowels as an intruder, is an unwelcome stranger, the parts are unused to it, like a crumb of bread which has gone the wrong way by passing into the lungs, and nature sets up a violent coughing to eject the intruder. As to the bowels, another plan is taken, but the object is the same—a speedy riddance. As soon as this unwelcome thing touches the lining of the bowels, nature becomes alarmed, and like as when a bit of sand is in the eye, she throws out water, as if with the intention of washing it out of the body, hence the sudden diarrheas with which persons are sometimes surprised. It was a desperate effort of nature to save the body, for if undigested food remains too long, either in the stomach or bowels, fits, convulsions, epilepsies, apoplexies, and death, are a very frequent result.

As a universal rule in health, and, with very rare exceptions, in disease, that is best to be eaten which the appetite craves or the taste relishes.

Persons rarely err in the quality of the food eaten; nature's instincts are the wise regulators in this respect.

The great sources of mischief from eating are three: Quantity, Frequency, Rapidity; and from these come the horrible dyspepsias which make of human life a burden, a torture, a living death.

RAPIDITY.—By eating fast, the stomach, like a bottle being filled through a funnel, is full and overflowing before we know it. But the most important reason is, the food is swallowed before time has been allowed to divide it in sufficiently small pieces with the teeth; for, like ice in a tumbler of water, the smaller the bits are, the sooner are they dissolved. It has been seen with the naked eye, that if solid food is cut up in pieces small as half a pea, it digests almost as soon, without being chewed at all, as if it had been well masticated. The best plan, therefore, is for all persons to thus comminute their food; for even if it is well chewed, the comminution is no injury, while it is of very great importance in case of hurry, forgetfulness, or bad teeth. Cheerful conversation prevents rapid eating.

Frequency.—It requires about five hours for a common meal to be dissolved and pass out of the stomach, during which time this organ is incessantly at work, when it must have re pose, as any other muscle or set of muscles, after such a length of effort. Hence persons should not eat within less than a five hours' interval. The heart itself is at rest more than one third of its time. The brain perishes without repose.

All are tired when night comes; every muscle of the body is weary and looks to the bed; but just as we lie down to rest every other part of the body, if we, by a hearty meal, give the stomach five hours' work, which, in its weak state, requires a much longer time to perform, than at an earlier hour of the day, it is like imposing upon a servant a full day's labor just at the close of a hard day's work; hence the unwisdom of eating heartily late in the day or evening; and no wonder it has cost many a man his life.

No laborers or active persons should eat an atom later than sun-down, and then it should not be over half the midday meal. Persons of sedentary habits, or who are at all ailing, should take absolutely nothing for supper beyond a single piece of cold stale bread and butter, or a ship-biscuit, with a single cup of warm drink. Such a supper will always give better sleep and prepare for a heartier breakfast, with the advantage of having the exercise of the whole day to grind it up and extract its nutriment.

Quantity.—It is variety which oftenest tempts to excess. Many a man has been about to push himself back from the table, with a feeling as if he did not want any more, when the unexpected appearance of some favorite dish has waked up a new appetite, and he "disposes" of an amount almost equal to that already taken. To prevent over-eating, take food deliberately, keep up a lively conversation on pleasurable subjects during the entire repast, and avoid a variety of dishes. For ordinary purposes, there should be on the family table but one kind of bread, one kind of meat, one kind of vegetable, one kind of drink, and one kind of fruit or berries, as dessert; butter, olive-oil, salads, cream, salt and pepper not being counted, but to be used as desired.

The most ruinous practice in reference to this subject is eating in a hurry, or under the influence of any disagreeable mental excitement, whether of anxiety, passion, or grief, for many have died within an hour by so doing.

Multitudes bring on themselves the horrors of a life-long dyspepsia by drinking large quantities of cold water at their meals, because by cooling the contents of the stomach, which maintains a heat of ninety-eight degrees, to that of the water drank at forty—ice-water being about thirty-two—digestion is as instantly arrested as a burning coal is extinguished by a dash of cold water; and this process is not resumed until heat enough has been drawn from the other parts of the body to raise the whole mass to its natural temperature; but this leaves the other parts of the system so cold that those who have not robust health sometimes rise from the table in a chill; at other times the general system, from want of vigor, has not been able to furnish the amount of heat necessary, digestion is not resumed, and diarrhea endangers life or convulsions destroy it within a few hours. Large quantities of hot drinks, at regular meals, will with equal certainty destroy the tone of the stomach and lay the foundation for tedious and painful diseases. Invalids should never take any cold drink at meals; and whether hot or cold, they are wise and safe who never allow themselves over a quarter of a pint of any liquid at a regular meal, or within an hour afterwards. A good position for the first half-hour after eating is either to stand or sit erect; better still, walk leisurely in the open air, if not too cold, or across the room with hands behind, chin a little elevated, maintaining an agreeable frame of mind. Particularly avoid a stooping position in sewing or reading for the first hour or two after meals, and also heavy lifting, hard study, or any intense mental emotion; these are all destructive of health; and although a single slight error may do no appreciable injury, it never fails to make an impress for ill, until at last there is one repetition too much, and a painful sickness, a life-long torture, or a speedy death from heart-disease, hemorrhage, or apoplexy winds up the sad history.

Never force food on the stomach. Never eat without an appetite. Never eat between meals.

Always take breakfast before leaving the house in the morning; this will prevent an easy and early tiring, while the testimony of observant farmers of education, corroborates the teachings of the best medical minds, that by strengthening the stomach and sending invigorating nutriment to the whole system, weakened by the long fast of the night, there is generated a power of resistance against the onsets of disease from the cold of winter and from the malarias and miasms of summer, especially in all flat, damp, and luxuriant soils, which can not be adequately expressed in language; while both experience and experiment have combined to show that by the simple expedient of an early breakfast, individuals and families and neighborhoods have exempted themselves from that scourge of all new countries, "Fever and Ague," especially if followed by a supper a little before sundown, from May to November.

### CATCHING COLD.

EXPERIENCED physicians in all countries very well know that the immediate cause of a vast number of cases of disease and death is a "cold;" it is that which fires a magazine of human ills; it is the spark to gunpowder. It was to a cold taken on a raw December day, that the great Washington owed his death. It was a common cold, aggravated by the injudicious advice of a friend, which ushered in the final illness of Washington Irving. Almost any reader can trace the death of some dear friend to a "little cold."

The chief causes of cold are two: first, cooling off too soon after exercise; second, getting thoroughly chilled while in a state of rest without having been overheated; this latter originates dangerous pleurisies, fatal pneumonias (inflammation of the lungs,) and deadly fevers of the typhoid type.

Persons in vigorous health do not take cold easily; they can do with impunity what would be fatal to the feeble and infirm. Dyspeptic persons take cold readily, but they are not aware of it, because its force does not fall on the lungs, but on the liver through the skin, giving sick-headache; and close questioning will soon develop the fact of some unusual bodily effort, followed by cooling off rapidly.

A person wakes up some sunny morning, and feels as if he had been "pounded in a bag;" every joint is stiff, every muscle sore, and a single step can not be taken without difficulty or actual pain. Reflection will bring out some unwonted exercise, and a subsequent cooling off before knowing it—as working in the garden in the spring-time; showing new servants "how to do;" in going a "shopping"—an expedition which taxes the mind and body to the utmost; the particular shade of a ribbon the larger or smaller size of a "figure" on a calico dress, or a camel's hair shawl; whether the main flower of a bonnet shall be "Jimpson" or a rose-bud; whether the jewelry shall sport a Cupid's arrow or a snake's head; these and similar debatable points on a thousand "little nothings," rouse women's minds to a pitch of interest and excitement scarcely excelled by that of counselors of state in determining the boundaries of empires or the fate of nations, to return home exhausted in body, depressed in mind, and thoroughly heated; the first thing done is to toss down a glass of water to cool off, next to lay aside bonnet, shawl, and "best dress," and lastly, to put on a cold dress, lie down on a bed in a fireless room, and fall asleep, to wake up with infinite certainty, to a bad cold, which is to confine to the chamber for days and weeks together, and not unseldom, carries them to the grave!

A lady was about getting into a small boat to cross the Delaware; but wishing first to get an orange at a fruit-stand, she ran up the bank of the river, and on her return to the boat found herself much heated, for it was summer, but there was a little wind on the water, and the clothing soon felt cold to her; the next morning she had a severe cold, which settled on her lungs, and within the year she died of consumption.

A stout, strong man was working in a garden in May; feeling a little tired about noon, he sat down in the shade of the house and fell asleep; he waked up chilly; inflammation of the lungs followed, ending, after two years of great suffering, in consumption. On opening his chest, there was such an extensive decay, that the yellow matter was scooped out by the cup ful.

A Boston ship-owner, while on the deck of one of his vessels, thought he would "lend a hand" in some emergency; and pulling off his coat, worked with a will, until he perspired freely, when he sat down to rest awhile, enjoying the delicious breeze from the sea. On attempting to rise, he found himself unable, and was so stiff in his joints, that he had to be carried home and put to bed, which he did not leave until the end of two years, when he was barely able to hobble down to the wharf on crutches.

A lady, after being unusually busy all day, found herself heated and tired toward sundown of a summer's day. She concluded she would rest herself by taking a drive to town in an open vehicle. The ride made her uncomfortably cool, but she warmed herself up by an hour's shopping, when she turned homeward; it being late in the evening, she found herself more decidedly chilly than before. At midnight she had *pneumonia*, (inflammation of the lungs,) and in three months had the ordinary symptoms of confirmed consumption.

A lady of great energy of character lost her cook, and had to take her place for four days; the kitchen was warm, and there was a draft of air through it. When the work was done, warm and weary, she went to her chamber, and lay down on the bed to rest herself. This operation was repeated several times a day. On the fifth day she had an attack of lung fever; at the end of six months she was barely able to leave her chamber, only to find herself suffering with all the more prominent symptoms of confirmed consumption; such as quick pulse, night and morning cough, night-sweats, debility, short breath, and falling away.

A young lady rose from her bed on a November night, and leaned her arm on the cold window-sill to listen to a serenade. Next morning she had *pneumonia*, and suffered the horrors of asthma for the remainder of a long life.

Farmers' wives lose health and life every year, in one of two ways; by busying themselves in a warm kitchen until weary, and then throwing themselves on a bed or sofa, without covering, and perhaps in a room without fire; or by removing the outer clothing, and perhaps changing the dress for a more common one, as soon as they enter the house after walking or working. The rule should be invariable to go at once to a warm room and keep on all the clothing at least for five or ten minutes, until the forehead is perfectly dry. In all weathers, if you have to walk and ride on any occasion, do the riding first.

An engineer, in the vigor of manhood, brought upon himself an incurable disease through a cold taken by standing on a zinc floor as soon as he left his bed in the morning, while he washed himself. Many a farmer's wife or daughter has lost her life by standing on a damp floor for hours together on washing-days.

A young lady, the only daughter of a rich citizen, stood an hour on the damp grass, while listening to the music in the Central Park; the next day she was attacked with inflammation of the lungs, of which she died within a week.

An estimable lady, a farmer's wife, busied herself in household affairs on a summer's day; late in the afternoon, having perspired a good deal, and being weary, she rode to town in an open vehicle to do some shopping; finding herself a little chilly, she walked rapidly on leaving her carriage, and soon became comfortably warm again. While shopping it rained. After the shower, she started homeward in a cool wind; this checked the perspiration the second time, and with all available precautions she reached home, chilled through and through, and died the victim of consumption within the year.

A farmer's daughter "went a-berrying;" the ground was flat and a little marshy; her shoes were thin, and by the excitement of company she remained several hours. She was ill next day. Four years later she stated to her physician that she had not seen a well hour since. She was then in the last stages of a hopeless decline, and died soon after.

A little attention would avert a vast amount of human suffering in these regards. Sedentary persons, invalids, and those in feeble health, should go directly to a fire after all forms of exercise, and keep all the garments on for a few minutes; or, if in warm weather, to a closed apartment, and, if any thing, throw on an additional covering. When no appreciable moisture is found on the forehead, the out-door garments may be removed. The great rule is, cool off very slowly always after the body has in any manner been heated beyond its ordinary temperature.

The moment a man is satisfied he has taken cold, let him do three things: First, eat nothing; second, go to bed, cover up warm in a warm room; third, drink as much cold water as he can, or as he wants, or as much hot herb-tea as he can; and in three cases out of four he will be almost well in thirty-six hours; if not, send for an educated and experienced physician at once, for any "cold" which does not "get better" within forty-eight hours, is neither to be trifled with nor experimented upon.

### DRESS.

The main object of dress is not to impart warmth, but to keep the natural warmth about the body; and thus prevent those sudden and fatal changes from heat to cold, which occur in passing from an in-door temperature of sixty-five degrees to that of zero or lower, without, as in mid-winter. The temperature of the Northern States varies over a hundred degrees during the year, sometimes nearly half that within twenty-four hours. Dress provides against these destructive sudden changes, by maintaining the warmth of the skin at its natural state, which is ninety-eight degrees, whether a man is on an iceberg in Greenland, or on a sand-island in a tropical sea. The materials of clothing which best keep the heat about the body are called *non*-conductors, such as furs and woolens, while the conductors are such as cool the body, by conveying the natural heat from it with great rapidity; the greater conducting ability is measured by the greater coldness which an article causes on the first instant of its application. In the very coldest weather, fur and woolen flannel appear but a little cool, and that but for an instant, and the next, there is a sensation of increasing, comfortable warmth; cotton flannel feels colder than woolen; silk colder than cotton; Irish linen colder than silk, and damp Irish linen greatly colder than either. A damp woolen shirt feels but a little cold, and begins to get warm and dry in an instant; even if the person is in a profuse perspiration; while an Irish linen or silk shirt, if damp with perspiration or otherwise, feels cold and clammy and sepulchral on the instant of its touching the skin, and will remain so for hours, without getting dry, never failing to leave a cold in some troublesome or even dangerous form; hence, as persons perspire easily and profusely in summer, Irish linen can not be worn in warm weather with impunity by the working classes, and those liable to perspiration from a little walking or exercise. Thus it is that British sailors in the navy are compelled to wear woolen flannel shirts all the year and in all latitudes; in the north, because it keeps the natural warmth from escaping from the body, thus maintaining a temperature of ninety-eight degrees about the skin; and in hot climates in summer, because although woolen is a bad conductor of heat, it is a good conductor of water, for if a woolen blanket is thrown over a sweating horse, in a very short time his hair and the inner side of the blanket will be dry, while the microscope will discover the whole outside surface spangled with millions of tiny drops of water. For these reasons, woolen flannel should be worn next the skin by all our people, from one year's end to another—a gauze material in summer; in winter, a more substantial article. White flannel fulls up, and becomes hard and stiff, unless about a fifth of it is cotton. Colored flannel, especially the red, always remains soft and pliable. These things are indisputably true, and a practical attention to them, on the part of all hard-working people, would prevent an amount of pain and sickness every year which figures can not express. This would be especially true, if in warm weather, when fires are not needed in the house, farmers and other laborers would wear a moderately stout article of red woolen flannel as a shirt, with nothing over it, while at work, but at other times a thin coat over that. Any flannel garment worn during the day should be hung up to air at night, while the night-gown all the year round should be of stout cotton shirting, for if woolen is worn next the skin all the time, it makes it callous, and is otherwise injurious. The best, safest, and most healthful head-dress for farmers and workmen, all the year round, is a common easy-fitting wool or felt hat; in winter it keeps the head warm, in summer it is a great protection against sun-stroke, especially if a silk handkerchief or a few leaves of a tree are worn in the crown; such a hat is a great preventive of baldness, if worn from early youth, because it allows the blood to flow freely to and from the scalp; but if the vessels are compressed, as is done by the common unyielding silk hat, the free circulation of the blood is obstructed, and the nourishment of the hair-roots or bulbs being cut off, the hair perishes irretrievably, causing all the discomforts and inconveniences of baldness.

Death often comes to the honest laborer, as well as to others, through the feet, either by tightly-fitting shoes, which, by obstructing the circulation, keep the feet cold, thus laying the foundation for troublesome diseases, or by shoes which do not keep out the dampness. In purchasing new shoes or having the measure taken, put on two pairs of woolen socks, without the knowledge of Crispin, and the new pair will feel from the first "as easy as an old shoe."

A piece of tarred or pitched cloth sewed between the layers of the shoe-sole is a great protection against dampness from without; or take pitch not hot enough to burn the leather, and apply it to the bottom and edges of the sole with a rag, let it dry thoroughly, and repeat the application thus three or four times; it is contended that a sole thus treated will not only be impervious to water and dampness, but will wear nearly twice as long as a sole not thus treated. It is an excellent plan to have two pairs of shoes, to be worn on alternate days, so as to have a perfectly dry pair to put on every morning, allowing the unworn ones to remain in a warm, dry place. Washing the feet every night in warm weather, and soaking them in warm water for ten minutes three times a week in winter, admirably promotes that warmth, pliability, and softness of the skin of the feet, so indispensable to health and comfort, saying nothing of the cleanliness of the practice, and its tendency both to prevent and to cure corns. But after all washings of the feet, it is of the first importance after wiping them well, to hold them to the fire and rub them with the hands until perfectly dry and warm in every part.

It will be useful to add here, in reference to corns, that they are caused by pressure and by friction also, hence they may be the result of a shoe that is either too tight or too loose. They can be always either permanently cured or kept within bounds by simply soaking the corn in hot water twenty minutes every night, and then patiently rub a few drops of sweet oil on the top of the corn; repeat the oil in the morning, and continue these until the core of the corn can be picked out with the finger-nail; nothing harder or sharper should ever touch a corn.

